# Changes in Biomarkers of Exposure and Potential Harm in Smokers Switched to Vuse Vibe or Vuse Ciro Electronic Nicotine Delivery Systems

**DOI:** 10.3390/toxics11070564

**Published:** 2023-06-29

**Authors:** Milly N. Kanobe, Paul R. Nelson, Buddy G. Brown, Peter Chen, Patrudu Makena, John W. Caraway, Gaddamanugu L. Prasad, Elaine K. Round

**Affiliations:** 1 RAI Services Company, 401 N. Main Street, Winston-Salem, NC 27101, USA; chenp@rjrt.com (P.C.); makenap@rjrt.com (P.M.); carawaj@rjrt.com (J.W.C.); 2 Former Employees of RAI Services Company, 105 Bowes Road, Winston Salem, NC 27106, USA; paul.nelson@mindspring.com; 3 Former Employees of RAI Services Company, 5714 Wonderous Lane, Durham, NC 27712, USA; brownb12@rjrt.com; 4 Former Employees of RAI Services Company, 490 Friendship Place Ct, Lewisville, NC 27023, USA; prasad.gaddamanugu@gmail.com; 5 Prasad Scientific Consulting LLC, 490 Friendship Place Ct, Lewisville, NC 27023, USA; 6 BAT (Investments) Limited, Globe House, 1 Water Street, London WC2R 3LA, UK; elaine_round@bat.com

**Keywords:** switching study, BoE, BoPH, cigarette smokers, abstinence, ENDS

## Abstract

Electronic nicotine delivery systems (ENDS) have the potential to provide nicotine to tobacco consumers while reducing exposure to combustion-related toxicants. Here, we report changes in biomarkers of exposure (BoE) and biomarkers of potential harm (BoPH) in smokers who completely switched to Vuse Vibe and Vuse Ciro ENDS products, or to smoking abstinence in a randomized, controlled clinical study. Thirteen BoE (12 urinary and one blood) that indicate exposure to harmful and potentially harmful toxicants (HPHCs) were evaluated at baseline on day 5. Urinary BoPH linked to oxidative stress, platelet activation, and inflammation were also assessed at baseline, and on day 5 and day 7. Nicotine exposure was lower in Vuse Vibe and Vuse Ciro groups compared to baseline values. Urinary non-nicotine BoE decreased significantly (52.3–96.7%) in the Vuse ENDS groups, and the reductions were similar in magnitude to those observed in the abstinence group. Blood carboxyhemoglobin decreased 52.8–55.0% in all study groups. Decreases (10–50%) in BoPH were observed in all study groups. Thus, smokers who switch exclusively to Vuse Vibe or Vuse Ciro products or completely abstain from smoking are exposed to substantially lower levels of HPHCs, and experience improvements in BoPH of oxidative stress and inflammation pathways.

## 1. Introduction

Cigarette smoking is a leading cause of preventable death, and significantly increases the risk of developing cancer and chronic heart and lung diseases [1]. Cigarette smoke contains thousands of compounds, including those designated as harmful and potentially harmful constituents (HPHCs) [2]. Chronic exposure to combustion-related toxicants causes persistent lung injury, oxidative stress, and chronic inflammation, which could progress to serious smoking-related diseases [1]. It is well established that complete cessation is the best option to reduce harm from cigarette smoking [3].

Other harm reduction approaches have recognized a “continuum of risk” among tobacco and nicotine products, whereby health risks are greatest for cigarette smoking and lowest for pharmaceutical nicotine replacement therapies [4,5]. Non-combustible tobacco products such as electronic nicotine delivery systems (ENDS) are estimated to be of significantly lower risk than cigarettes, and are therefore positioned at the lower end of the risk continuum [6]. ENDS are a heterogeneous class of alternative nicotine delivery products and have gained significant market presence in the past decade [7,8]. These products deliver nicotine to smokers with minimal exposure to combustion-related toxicants [9], and hence could reduce harm from cigarette smoking.

Typically, ENDS consist of a rechargeable power unit and use disposable or reusable cartridges containing e-liquids of varying nicotine contents and flavors. Because ENDS produce aerosol by heating the e-liquid rather than burning tobacco, the resulting aerosol from ENDS use is less complex than cigarette smoke. Several laboratories have demonstrated that ENDS aerosol, relative to cigarette smoke, contains far fewer known toxicants and at significantly lower levels [10,11,12]. Consequently, ENDS users are exposed to fewer and substantially lower levels of HPHCs than smokers [11,13,14,15]. Overall, ENDS are thought to have the potential to reduce the public health impact of smoking by reducing the risk of smoking-related disease [16,17,18]. Given that ENDS are a vastly diverse class of products, there is a need for a rigorous evaluation of the products, and in general, the long-term health effects of ENDS are an active area of research.

In the United States, marketing of all new tobacco products, including ENDS, must be authorized by the US Food and Drug Administration (FDA) through the Premarket Tobacco Product Application (PMTA) pathway. The marketing of the new tobacco products must be shown to be appropriate for the protection of public health and must consider the risks and benefits to the population as a whole, including individual users and nonusers of the tobacco product [19]. A key component in the evaluation of the individual health impact of ENDS is the determination of exposure to toxicants and the effects of exposure through biomarkers of exposure (BoE) and biomarkers of potential harm (BoPH), respectively. A set of 20 BoE, representing different classes of HPHCs, is used to measure exposure to tobacco products [20]. The BoE evaluated in this study represent constituents for which validated biomarkers exist and that were recommended for evaluation in e-liquids and aerosols by the FDA in the 2016 [21] and 2019 [19] ENDS PMTA documents, and/or constituents identified by the FDA as HPHCs in tobacco products or tobacco smoke [2].

BoPH are a class of early clinical risk markers, and encompass several different biological pathways known to play a role in smoking-related diseases such as chronic heart disease and chronic respiratory disease [22]. A majority of BoPH map to oxidative stress and inflammation pathways, which are involved in the progression toward smoking-related diseases [1,22]. For example, arachidonic acid metabolites such as isoprostanes, thromboxane A_2_ metabolites (as markers of oxidative stress), and leukotriene E_4_ (LTE_4_) and white blood cell counts (markers of inflammation) are elevated in chronic healthy smokers compared to non-smokers [23,24], and have been used to assess physiological responses to changes in smoking status [25,26,27].

Previously, we reported changes in BoE and BoPH in smokers switched to the Vuse Solo digital vapor system, a first-generation cigalike ENDS, in original and menthol flavors [26,28,29,30]. Here, we report changes in BoE and BoPH over 5- and 7-day periods in smokers who used two different closed ENDS products (Vuse Vibe Original and Vuse Ciro Original, both with tobacco-flavored e-liquids). These tobacco-flavored variants of the two Vuse ENDS products (Ciro and Vibe) have received Marketing Granted Orders from the FDA through the PMTA process [31,32]. The results described in this manuscript formed part of the body of scientific evidence included in the PMTAs for Vuse Vibe and Vuse Ciro, making them of interest to the scientific and tobacco research community.

## 2. Materials and Methods

### 2.1. Study Design

The clinical study aimed to investigate biomarker changes occurring in the blood and urine samples of smokers switched to one of three different Vuse ENDS products marketed by the R.J. Reynolds Vapor Company (Winston-Salem, NC, USA). Study findings regarding Vuse Vibe and Vuse Ciro products are presented in this manuscript, whereas the results relating to a third product (Vuse Solo) were recently published [30]. This two-center, randomized, controlled, switching, open-label, parallel group, clinical confinement study was conducted at two US clinical research sites (ClinicalTrials.gov identifier number; date: NCT03170674; 31 May 2017). Ethical approval was obtained from MidLands Independent Review Board, (Overland Park, KS, USA), and written informed consent was obtained from all study participants prior to study participation.

The primary objective of the clinical study was to assess changes in biomarkers of exposure (BoE) after a 5-day, in-clinic switch from usual brand (UB) cigarettes to use of Vuse Vibe or Vuse Ciro ENDS, or to Abstinence. Secondary objectives included assessments of changes in urinary total nicotine equivalents and daily ENDS product use amounts, as well as evaluation of urinary arachidonic acid (AA)-derived metabolites as BoPH on day 5 and day 7 after switching from UB cigarettes to use of Vuse ENDS or to smoking abstinence.

Several previous studies, including ours, have shown that HPHCs, as measured by BoE levels, markedly decline within 3–5 days of smoking abstinence due to their short-half-lives [29,30]. Since the adverse effects of smoking, however, can persist for several months to years and are not immediately reversible following smoking abstinence, we have sought to identify BoPH that rapidly respond to changes in smoking abstinence. Based on our previous work, we measured BoPH after 5 and 7 days of smoking abstinence or switching to the Vuse study products.

This clinical study was conducted under clinical confinement conditions to ensure compliance with the study protocols and ensure that the subjects do not use non-study products. The initial 2 days of clinical confinement following enrollment was necessary to allow acclimatization of subjects to the clinical site, collection of samples at baseline, and randomization to the study products. The subjects were switched to abstinence, or to either Vuse Ciro or Vuse Vibe ENDS product use for 7 days, meaning a total confinement period of 10 days.

### 2.2. Study Products

Vuse Vibe and Vuse Ciro products are pre-filled, closed ENDS products (RJR Vapor Company, Winston-Salem, NC, USA) that were commercially available at the time the study was conducted (Supplementary Table S1). Both Vuse Vibe and Vuse Ciro were used with Original, tobacco-flavored, nicotine containing e-liquids. The Vuse Ciro product was originally marketed under the brand name “Vuse Solo+”. The product was subsequently renamed to “Vuse Ciro”.

The Vuse Vibe power unit is powered by a ≥550 -mAh rechargeable battery and is paired with a cartridge containing 1.9 mL of e-liquid with 3% nicotine content by weight (36 mg/mL) and a propylene glycol-to-glycerin ratio (PG/VG) of 20/80. The maximum puff duration for Vuse Vibe is 6 s. The Vuse Ciro power unit has a ≥260 -mAh battery and uses a cartridge containing 0.9 mL of e-liquid with 1.5% nicotine by weight (17.7 mg/mL) and a PG/VG ratio of 29/71. The maximum puff duration for Vuse Ciro is 10 s.

### 2.3. Study Conduct

Generally healthy adult smokers, meaning men and women aged 21–60 years who had smoked at least 10 combustible filtered non-menthol cigarettes per day for at least 6 months prior to screening were recruited into the study. Tobacco-flavored ENDS were chosen for evaluation in this study. In order to maintain the flavor preference of the smokers, menthol smokers were excluded, and only non-menthol smokers were included in the study. In total, 127 participants were enrolled into the study. Details about participant disposition into the study are given in the Results section, under data sets analyzed. Full inclusion and exclusion criteria are detailed in the Supplementary Material, and subject demographics are summarized in Supplementary Table S2.

**Figure 1:**
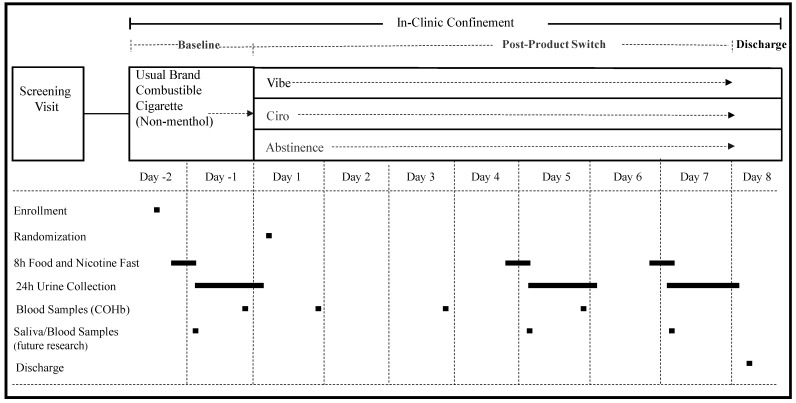
**Key events and timeline of the Vuse ENDS clinical study**: Following randomization, enrolled subjects were assigned to using Vuse Vibe, Vuse Ciro, or smoking abstinence. Blood and urine samples were collected for biomarker analyses at baseline and Day 5 (BoE) and BoPH (Days 5 and 7).

Potential participants completed a pre-screening telephone interview and a screening visit to assess their eligibility within 30 days prior to study entry (i.e., prior to day −2) (Figure 1). On day −2, eligible participants were enrolled in the study and confined at the clinical site for 10 days. Baseline assessments during *ad libitum* smoking of their UB cigarettes (supplied by study participants themselves) occurred in the first 2 days (day −2 and day −1). On day 1, the participants were randomized to the Vuse Solo, Vuse Vibe, Vuse Ciro or Abstinence groups. *Ad libitum* use of Vuse ENDS products was allowed between 700 h and 2300 h on Days 1–7.

### 2.4. Biomarkers

Biomarkers of constituents representing different classes of HPHCs are commonly used to assess exposure to cigarette smoke [20]. The FDA has published an established list of HPHCs in tobacco products [2]. The biomarkers of exposure chosen included all constituents from that list that have a representative biomarker of that class of compounds and have been shown to distinguish between smokers and non-smokers in previous studies (papers described in [29]). We selected a subset of 14 constituents with representative BoE and measured them in urine and blood (Supplementary Table S3). Thirteen of these BoE were measured in 24 h urine samples collected from the morning of day −1 to the morning of day 1 (baseline), and again on the morning of day 5 through the morning of day 6. Total daily urinary amounts excreted (total mass/day) were determined by multiplying the concentrations by the collected 24 h urine sample volume (mass/mL × volume in mL) [29]. Exposure to nicotine is reported as a composite measure of total nicotine equivalents (NicEq-T). The NicEq-T represent about 90% of nicotine exposure and were measured in urine (Supplementary Table S3) [33,34]. Blood samples for the measurement of COHb were collected approximately 12 h after the start of product use on days −1, 1, 3, and 5. The BoE were quantified using established methods [30,35,36,37,38,39], which are detailed in the Supplementary Materials.

The 24 h urine samples for the assessment of changes in BoPH were collected on study days −1, 5, and 7.

All samples for BoE and BoPH were analyzed using validated methods at a qualified bioanalytical laboratory, under good laboratory practices quality control (Supplementary Table S3) [29]. A panel of urinary AA metabolites (LTE_4_, 2,3-dinor thromboxane B2 [2,3-d-TXB_2_], and 11-dehydro-thromboxane B_2_ [11-dh-TXB_2_]) was assessed as BoPH, as described previously [26]. The selection of the BoPH for evaluation in this study was based on their ability to respond to changes in smoking status within the short duration (7 days) of the study period. Published works from this and other research groups have shown that these three BoPH respond rapidly and show improvement when smokers abstain for short periods (few days), or exclusively use other non-combustible tobacco products. Based on those findings [26], the three BoPH were selected for evaluation in this study.

### 2.5. Daily Vuse ENDS Product Consumption

The participants’ daily usage of Vuse ENDS products was measured as the mass (grams) of e-liquid used per day (Supplementary Table S4). This was achieved by weighing the unused cartridge before assembling and dispensing to the participant and weighing the used cartridge upon the participant returning the ENDS product after use (disassembled from the power unit). The difference between the initial (unused cartridge) and the end (used cartridge) weight was recorded as the daily ENDS product usage, and the sum of the differences in weight of individual participants’ used cartridges on each day of use constituted the daily Vuse ENDS product use amounts.

### 2.6. Statistical Analysis

Sample size calculation was based on 3-hydroxy-benzo(a)pyrene (3-OH-B[a]P), a BoE with high variability but a small effect size in smokers switching to ENDS [29,40]. It was estimated that 31 participants per Vuse ENDS group would be sufficient to detect at least a 65% reduction in overall 3-OH-B[a]P concentration with 80% power, using a Bonferroni-adjusted significance level (α = 0.0038, calculated as 0.05/13 for the number of comparison tests). Thus, the aim was to enroll 35 participants per Vuse ENDS group. No formal sample size calculation was performed for the abstinence group, but based on previous experience, we estimated that enrolling 15 participants to achieve 10 participants completing the study would be sufficient to observe a decrease in BoE at 5 days after abstaining from smoking. All participants who completed the study were included in the BoE analyses. Incomplete data were treated as missing data.

To account for multiplicity and to preserve the overall type I error rate of 0.05, each test was adjusted using the adaptive step-down Bonferroni method implemented in SAS version 9.4 (SAS Institute, Cary, NC, USA) [41]. No multiplicity adjustments were made for the assessment of NicEq-T and BoPH. We set the significance threshold at *p* = 0.05, calculated with a one-sided paired *t*-test for the primary BoE and BoPH, and a two-sided paired *t*-test for NicEq-T. Descriptive statistics and mean (SD) percentage changes from day −1 (baseline) to day 5 after switching were calculated from raw data. No comparisons were made between study groups.

## 3. Results

### 3.1. Data Sets Analyzed

The clinical study enrolled a total of 127 participants, and 2 participants voluntarily withdrew consent prior to randomization. Of the 125 randomized subjects, the following assignments were made: 37 to the Vuse Vibe group, 37 to the Vuse Ciro group, and 16 to the Abstinence group. Thirty-five participants were randomized to the Vuse Solo group, and those results are reported separately [30]. Among the participants randomized to Vuse Vibe, Vuse Ciro or Abstinence groups, 79 (87.8%) completed the study through to study day 7 (Vuse Vibe group, *n* = 33; Vuse Ciro group, *n* = 35; Abstinence group, *n* = 11).

### 3.2. Demographics and Product Use History

The demographic and baseline characteristics of participants were similar among the study groups (Supplementary Table S2). Overall, 58 (64.4%) participants were male and 32 (35.6%) were female. The mean age of participants was approximately 40 years, and the majority (72.2%) were Caucasian. Most of the study participants (94.4%) were predominantly non-Hispanic/Latino. The average number of years of smoking and the average number of cigarettes smoked per day by all participants were comparable across the study groups.

### 3.3. Biomarkers of Exposure in Urine and Blood

**Figure 2:**
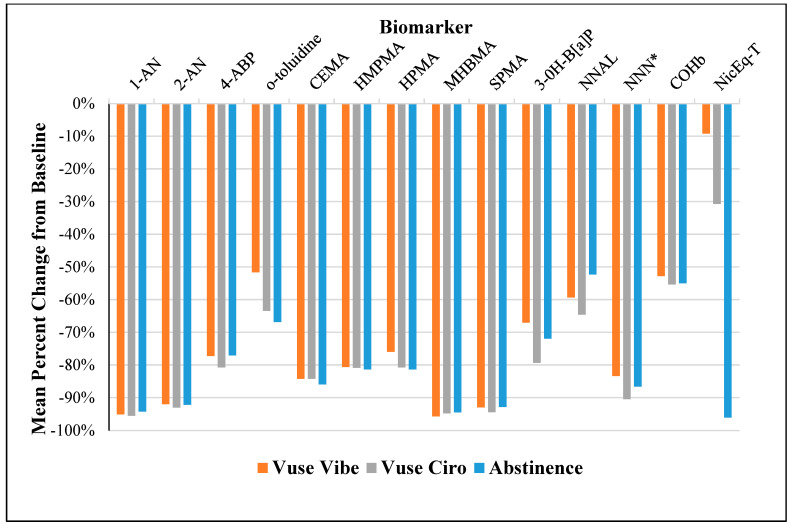
**Mean percent change in BoE in smokers switched to Vuse Vibe or Vuse Ciro products:** Biomarkers of Exposure decline in smokers who switched to Vuse Vibe, Vuse Ciro or Abstinence for 5 days. Carboxyhemoglobin is a blood biomarker. Exposure to nicotine was determined by quantifying nicotine and its 5 metabolites, and reported as Total Nicotine Equivalents (NicEq-T). The BoE are indicated in the parentheses for the HPHCs evaluated, along with their acronyms: 1-AN (1-aminonaphthaline); 2-AN (2-aminonaphthalene); 4-ABP (4-aminobiphenyl); Acrylonitrile (2-cyanoethyl mercapturic acid [CEMA]); Crotonaldehyde (3-hydroxy-1-methylpropylmercapturic acid [HMPMA]; ); Acrolein (3-hydroxypropyl mercapturic acid [HPMA]); 1,3-Butadiene (Monohydroxybutenyl mercapturic acid [MHBMA]); Benzene (S-phenyl mercapturic acid [SPMA]); 3-OH-B[a]P (3-hydroxy-benzo[a]pyrene); NNK: (Nicotine-derived nitrosamine ketone; 4-(methylnitrosamino)-1-(3-pyridyl)-1-butanol + glucuronides [Total NNAL]); NNN (N-nitrosonornicotine; N′-nitrosonornicotine + glucuronides [Total NNN]); COHb: carboxyhemoglobin. * Excluding participants with extreme data (data that were beyond the arithmetic mean ± 6 multiplied by standard deviation for each study endpoint).

Mean urinary NicEq-T from baseline (day −1) to day 5 indicated reductions in nicotine uptake during Vuse ENDS product use, with statistically non-significant reductions in the Vuse Vibe group (9.21%; *p* = 0.29), and statistically significant reductions observed in the Vuse Ciro group (30.8%; *p* = 0.0004) compared to baseline concentrations. NicEq-T was statistically significantly decreased by 96.1%; *p* < 0.0001 in the Abstinence group compared to baseline values (Figure 2; Table 1).

All of the non-nicotine BoE were statistically significantly decreased (*p* ≤ 0.001; 55–96%) from baseline among each study group, five days after switching from UB cigarettes to Vuse Vibe, Vuse Ciro, or Abstinence (Table 1). Mean percent reductions in non-nicotine BoE were generally similar among the study groups (Figure 2). Acronyms of the HPHCs and corresponding BoE are provided in the caption of Figure 2 and in Supplementary Table S3.

Table 1: Mean percent (%) change in BoE from baseline to day 5 after the Vuse product switch. Statistically significant changes were detected in all the biomarkers tested in the Vuse Vibe and Vuse Ciro groups by day 5, relative to their baseline values, with the exception of NicEq-T in the Vuse Vibe group (* not statistically significantly different). These BoE declines were similar to those measured in the Abstinence group, except for NicEq-T levels, which were more modest in the Vuse ENDS groups, which is consistent with expectation of smokers switched to a nicotine-containing product. Acronyms of the HPHC and corresponding BoE are provided in the legend of Figure 2 and Supplementary Table S2. Statistical significance for biomarker changes was calculated as described in Materials and Methods. These reductions in the BoE are reflective of markedly reduced exposure to the respective classes of HPHCs when smokers are completely switched to Vuse Vibe or Vuse Ciro. For example, mean levels of BoE of tobacco-specific nitrosamines (NNAL and NNN), polycyclic aromatic hydrocarbons (B[*a*]P), volatile organic compounds (HPMA, HMPMA, MHBMA, CEMA and SPMA), and aromatic amines (1-AN, 2-AN, 4-ABP and *o*-Tol) had decreased statistically significantly in the Vuse users by day 5, relative to their mean baseline values, and these reductions were similar in magnitude to those observed in the Abstinence group.

Blood carboxyhemoglobin levels were statistically significantly decreased (*p* < 0.05) from baseline to study day 5 in both the Vuse ENDS and Abstinence groups (Table 1). The percent reductions in mean COHb levels from baseline to day 5 were 52.8%, 55.4% and 55.0% for the Vuse Vibe, Vuse Ciro, and Abstinence groups, respectively.

**Table 1:** Mean percent (%) change in biomarkers of exposure from baseline to day 5 post Vuse product switch.

Biomarker of Exposure	Vuse Vibe			Vuse Ciro			Abstinence		
	Day −1	Day 5	% Change	Day −1	Day 5	% Change	Day −1	Day 5	% Change
Total nicotine equivalents (mg/24 h)	17.46 ± 6.93	15.85± 10.53	–9.21	16.26 ± 7.69	11.26 ± 8.54	–30.76	16.79 ± 8.80	0.65 ± 1.10	–96.12
Total NNAL (ng/24 h)	559.66 ± 333.35	227.58 ± 152.39	–59.34	710.19 ± 413.61	251.62 ± 165.43	–64.57	600.59 ± 404.24	286.51 ± 166.15	–52.30
Total NNN (ng/24 h)	12.82 ± 14.12	2.13 ± 0.99	–83.40	20.18 ± 40.27	1.93± 0.95	–90.46	63.45 ± 165.86	2.11 ± 0.80	–96.68
3-OH-B[a]P (pg/24 h)	366.40 ± 471.00	100.91 ± 172.60	–72.46	317.52 ± 352.53	65.54 ± 52.80	–79.36	218.70 ± 217.70	61.38 ± 43.61	–71.94
CEMA (µg/24 h)	256.23 ± 88.46	40.36 ± 16.19	–84.25	259.49 ±109.90	40.84 ± 18.77	–84.26	245.87 ± 125.18	34.57 ± 22.44	–85.94
HMPMA (µg/24 h)	536.87 ± 211.63	104.13 ± 50.79	–80.60	540.67 ± 259.49	103.50 ± 76.24	–80.76	521.64 ± 245.67	97.18 ± 53.90	–81.37
HPMA (µg/24 h)	1622.17 ± 495.92	389.7 8± 166.93	–75.97	1710.51± 786.41	329.71 ± 98.00	–80.72	1634.71 ± 997.90	304.58 ± 106.54	–81.37
MHBMA (ng/24 h)	3209.01 ± 2114.75	137.35 ± 63.56	–95.72	2461.32 ± 2039.07	127.44 ± 57.91	–94.82	2474.69 ± 1803.87	135.83 ±51.83	–94.51
SPMA (ng/24 h)	5276.70± 2679.05	368.72 ± 180.72	–93.01	5352.37 ± 4019.57	296.70 ± 153.79	–94.46	4702.19 ± 3151.04	335.61 ± 214.66	–92.86
1-AN (ng/24 h)	104.34 ± 38.17	5.07 ± 4.32	–95.14	106.97 ±54.80	4.76 ± 5.21	–95.55	93.27 ± 55.58	5.34 ±3.55	–94.28
2-AN (ng/24 h)	26.04 ± 12.67	2.09 ± 1.01	–91.99	28.66 ± 17.01	1.99 ± 1.12	–93.06	27.55 ± 14.39	2.15 ± 1.01	–92.20
4-ABP (ng/24 h)	20.14 ± 8.39	4.58 ± 2.22	–77.28	22.48 ± 22.43	4.33 ± 2.58	–80.75	19.45 ± 9.32	4.46 ± 2.45	–77.07
*o*-Toluidine (ng/24 h)	192.93 ± 66.36	93.35 ± 54.06	–51.62	212.94 ± 94.77	77.83 ± 48.56	–53.45	204.36 ± 125.18	67.70 ± 25.90	–66.70
COHb (%)—blood	11.33 ± 3.01	5.35 ±0.96	–52.79	11.68 ± 3.44	5.21 ± 0.89	–55.36	11.73 ± 3.95	5.28 ± 1.10	–55.01

### 3.4. Biomarkers of Potential Harm

The results summarizing changes in three AA metabolites (LTE_4_, 2,3-d-TXB_2_, and 11-dh-TXB_2_) as BoPH, relative to the baseline, are presented in Table 2. Overall, there were consistent decreases in the BoPH in smokers who were switched to the investigational products, and those changes are comparable with participants who abstained from smoking.

Participants who switched to smoking abstinence showed statistically significant decreases in urinary levels of LTE_4_ on day 5 (48%) and on day 7 (35%), 2,3-d- TXB_2_ on day 5 (53%) and on day 7 (35%), and 11-dh-TXB_2_ on day 7 (28%). Although 11-dh-TXB_2_ levels on day 5 were lower (21%), these values were not statistically significantly different from baseline.

Participants switched from combustible cigarettes to Vuse Ciro experienced statistically significant decreases in urinary levels of LTE_4_ on day 5 (32%) and on day 7 (39%), 2,3-d-TXB_2_ on day 5 (48%), and 11-dh-TXB_2_ on day 7 (20%). Statistically non-significant decreases in 2,3-d-TXB_2_ on day 7 (39%) and 11-dh-TXB_2_ on day 5 (18%) were observed in Vuse Ciro users.

Participants switched from combustible cigarettes to Vuse Vibe experienced statistically significant decreases in urinary levels of LTE_4_ on day 5 (30%) and on day 7 (38%). Decreases in the levels of 2,3-d- TXB_2_ on days 5 (22%) and 7 (24%) in Vuse Vibe users, however, were non-significant. The levels of 11-dh-TXB_2_ were statistically significantly lower on days 5 (10%) and 7 (25%) among those who were switched to Vuse Vibe.

Thus, the changes in BoPH levels in the smokers who were switched to Vuse Vibe or Ciro were directionally concordant and comparable in magnitude with those in the Abstinence group.

**Table 2:** Changes in biomarkers of potential harm in smokers switched to Vuse Vibe or Vuse Ciro products.

		Mean (SD)			*p*-Value from Paired		Change in BoPH Levels	
					*t*-Test			
BoPH	Cohort	Baseline	Day 5	Day 7	Baseline vs. Day 5	Baseline vs. Day 7	Baseline vs. Day 5	Baseline vs. Day 7
(ng/24 h)								
LTE_4_	Abstinence	111.5 (58.0)	58.2 (38.1)	72.7 (57.4)	0.001	0.032	−48%	−35%
	Ciro	134.3 (63.0)	90.8 (43.8)	81.9 (33.3)	<0.001	<0.001	−32%	−39%
	Vibe	128.0 (55.9)	89.6 (38.2)	79.0 (32.8)	<0.001	<0.001	−30%	−38%
2,3-d-TXB_2_	Abstinence	937.1 (804.3)	444.4 (393.7)	625.0 (437.3)	0.041	0.044	−53%	−33%
	Ciro	905.3 (788.5)	475.0 (355.8)	548.3 (580.7)	0.004	0.06	−48%	−39%
	Vibe	774.7 (823.7)	605.9 (432.6)	585.1 (530.1)	0.089	0.053	−22%	−24%
11-dh-TXB_2_	Abstinence	492.8 (200.7)	388.2 (238.8)	355.4 (217.2)	0.121	0.017	−21%	−28%
	Ciro	452.6 (177.0)	372.0 (128.2)	360.0 (123.0)	0.053	0.013	−18%	−20%
	Vibe	477.5 (243.4)	431.1 (181.0)	360.2 (134.8)	0.04	<0.001	−10%	−25%

### 3.5. Vuse ENDS E-Liquid Consumption

Participants used their randomized study products over the 7-day study period. Following product switching, the mean daily amounts of Vuse Vibe and Vuse Ciro e-liquid (g) used steadily increased from day 1 to day 7. The mean daily e-liquid use increased from 0.82 to 1.29 g in the Vuse Ciro group, and from 1.00 to 1.76 g, in the Vuse Vibe group over the 7 days. The mean number of cartridges used daily was 0.72 ± 0.36 for Vuse Vibe and 1.76 ± 1.01 for Vuse Ciro (Supplementary Table S4).

### 3.6. Adverse Events

In total, 81 adverse events (AEs) were reported by 47 participants: 36 among 17 participants in the Vuse Vibe group, 32 among 20 participants in the Vuse Ciro group, and 13 among 10 participants in the Abstinence group.

The most frequently reported AEs in the Vuse Vibe group were headache, cough, and nausea (four cases each), and back pain and oropharyngeal pain (three cases each). Other less frequently reported AEs (one case per AE) in the Vuse Vibe group were abnormal dreams, arthralgia, bacterial vaginosis, chest discomfort, constipation, dry mouth, ecchymosis, hemoptysis, muscle spasms, musculoskeletal pain, neck pain, palpitations, paraesthesia, presyncope, respiratory tract congestion, sneezing, and upper respiratory tract infection.

Subjects randomized to the Vuse Ciro group most often reported the following AEs: headache and oropharyngeal pain (three cases each); cough, upper respiratory tract infection, wheezing, and Lhermitte’s sign (two cases each). Other less frequently reported AEs (one case per AE) by the subjects in Vuse Ciro group included abdominal discomfort, acrodermatitis, agitation, appetite disorder, arthralgia, dizziness, dry mouth, dry throat, musculoskeletal pain, myalgia, pain in extremities, paraesthesia, paraesthesia oral, pollakiuria, presyncope, rash, rhinitis, and vulvovaginal candidiasis.

Subjects in the Abstinence group most often reported irritability and back pain AEs (two cases each). Other less frequently reported AEs (one case per AE) in the Abstinence group were affect lability, cough, ear discomfort, feeling abnormal, headache, hypoacusis, insomnia, musculoskeletal pain, and restless leg syndrome.

Seventy-two (89%) AEs were mild, nine (11%) were moderate, and none were severe in severity. The severity of the AEs was categorized as mild (of little concern to the participant and/or of no clinical significance, and not expected to have any effect on the participant’s health or well-being), moderate (discomfort enough to cause interference with or change in usual activities, and likely to require medical intervention and/or close follow-up), and severe (incapacitating, leaving the subject unable to work or participate in many or all usual activities, being of concern to the subject and/or posing substantial risk to the participant’s health or well-being, and likely to require medical intervention and/or close follow-up).

Some 40 (56%) of the 81 AEs (Vuse Vibe, *n* = 19; Vuse Ciro, *n* = 21) were determined by the principal investigator to be associated with the use of the study products. Three participants discontinued the study due to AEs (Vuse Vibe, *n* = 1 due to mild low back pain and bacterial vaginosis; Vuse Ciro, *n* = 1 due to moderate acrodermatitis; Abstinence, *n* = 1 due to moderate feeling abnormal), none of which were deemed to be related to the investigational product use. Supplementary Table S5 summarizes the AEs reported during the study in each of the study groups.

Table 2: Changes in biomarkers of potential harm in smokers switched to Vuse Vibe or Vuse Ciro products: baseline, day 5 and day 7 levels of the BoPH (mean [standard deviation]) were measured in the 24 h urine samples of smokers who switched to Vuse Ciro, Vuse Vibe or Abstinence. Statistical significance is defined as *p* ≤ 0.05. Mean % changes from the baseline values are also presented.

## 4. Discussion

Chronic exposure to HPHCs in cigarette smoke perturbs numerous biological pathways and causes diseases. Here, we demonstrate that smokers who completely switch to Vuse Vibe or Vuse Ciro ENDS products for 5 days significantly reduce their exposure to several representative HPHCs to levels comparable to those observed with smoking abstinence, as measured by the changes in BoE. Further, switching to the Vuse ENDS products or complete abstinence results in similar decreases in three BoPH of oxidative stress and inflammation.

Consistent with FDA’s recommendations for PMTAs at the time the study was conducted, we evaluated several BoE indicative of exposure to HPHCs in smokers who were switched exclusively to Vuse Vibe or Vuse Ciro ENDS product use for 5 days. Mean urinary NicEq-T decreased in the two Vuse groups (9.21% and 30.8% for Vibe and Ciro, respectively) reflecting the exposure to nicotine from the use of the investigative products (Table 1). The Vuse Vibe product has a higher nicotine level (3%) compared to the Vuse Ciro product (1.5% nicotine by weight), and the nicotine levels observed in this study seem to trend with the nicotine levels in the products.

Substantial decreases in BoE from baseline smoking were evident in the Vuse product groups as well as the Abstinence group (Table 1; Figure 2). Exposure to TSNAs was lower in the Vuse ENDS and Abstinence groups; for example, NNK exposure decreased by 50–65%, reflecting its longer half-life [33] compared to NNN, which decreased by 80–97%. The levels of 3-hydroxy-benzo[a]pyrene, representing exposure to polycyclic aromatic hydrocarbons, decreased by 67–80%. The BoE of volatile organic compounds (CEMA, SPMA, MHBMA, HPMA, HMPMA, and aromatic amines 1-AN, 2-AN, 4-ABP, and *o*-toluidine*)* were also substantially lower in all the three study groups. Overall, these BoE declines are consistent with the findings from studies regarding Vuse Solo, one study in which menthol and non-menthol products were tested [29], and the other wherein only non-menthol products were evaluated [30]. Further, similar reductions in nicotine exposure, TSNAs, and CEMA levels were reported in smokers who abstained from smoking for 7 days in a smoking abstinence biomarker study [42].

The findings in this study are also consistent with findings of other researchers showing that BoE levels in ENDS product users are lower than in smokers [11,13,43,44,45]. In addition to short-term switching studies (as reported here), some investigators have reported decreases in select BoE in smokers switched to exclusive ENDS use for at least six months [27]. Importantly, data from the large population-based Population Assessment of Tobacco and Health study reveal that smokers switched to exclusive ENDS use are exposed to significantly lower amounts of HPHCs [46]. Thus, the marked reductions in BoE observed in this 5-day confinement study are expected to be sustained in exclusive Vuse ENDS product users in real-world settings.

Since smoking-related diseases are multifactorial and develop over several decades of sustained exposure to smoke toxicants, BoPH are useful mechanistic, interim markers that could inform clinical risk [4,22]. In efforts to identify short-term BoPH that respond to changes in smoking status, we previously reported that urinary eicosanoids, LTE_4_, 11-dh-TXB_2_ and 2,3-d-TXB_2_ were significantly elevated in smokers, compared to smokeless tobacco users and non-tobacco users [24]. Among these BoPH, LTE_4_, and 2,3-d-TXB_2_ were shown to rapidly decrease upon smoking abstinence or switching to non-combustible tobacco products [26]. LTE_4_ is a marker of inflammation and a measure of total cysteinyl leukotrienes, which are associated with several diseases including asthma [47]. Consistent with our previous work, we find that switching smokers to Vuse ENDS investigational products or abstinence results in decreases in the levels of LTE_4_ [30] (Table 2).

Thromboxane A2, an atherogenic and a vasoconstrictive agent, is generated from arachidonic acid, which promotes platelet aggregation in response to endothelial injury and contributes to cardiovascular diseases [48]. The metabolites of thromboxane A_2_, 11-dh-TXB_2_, and 2,3-d-TXB_2_ are widely used as markers of platelet activation [49,50]. These two metabolites were reported to be elevated in smokers compared to non-smokers [23,51,52]. The levels of 2,3-d-TXB_2_, although consistently lower across the study groups versus baseline, were statistically significant only in the abstinence group. The levels of 11-dh-TXB_2_ were also consistently lower, and reached statistical significance after 7 days of switching to Vuse Ciro or smoking abstinence. While the BoPH consistently declined upon smoking cessation or switching to the Vuse products for 5 and 7 days, in some instances, the decreases were smaller in magnitude on day 7 compared to day 5. Other studies, which reported decreases in 11-dh-TXB_2_ levels upon switching to different inhaled, noncombustible tobacco products, were longer (90–360 days) in duration [25,27,53,54]. Thus, the levels of all three BoPH in the product use groups were comparable to those in the Abstinence group, and were suggestive of favorable biological changes resulting from reduced exposure to cigarette smoke toxicants.

Among the limitations of this study is the lack of a parallel smoking group as an additional control. This would have allowed for the evaluation of BoE differences in the continuous smoking group as well as the Ciro, Vibe or Abstinence group under same evaluation conditions. Instead, the current study focused on within-subject changes in biomarkers in this short-term switching study. The design of this study provided an assessment of changes in BoE and BoPH under the conditions of a short-term switch, and as such provides directional but limited information on likely longer-term changes and the potential health benefits of switching to ENDS. Others have reported sustained decreases in BoE in exclusive users of ENDS after 6+ months, following 10 years of cigarette smoking [27], consistent with the short-term trends observed in the current study. While we explored a limited number of BoPH in this manuscript, a broader, longitudinal investigation would further improve our understanding of the health impact of ENDS.

## 5. Conclusions

Since combustion-related toxicants are the drivers of smoking-related diseases, reducing exposure to these toxicants by switching to alternate non-combustible tobacco products is a key element of tobacco harm reduction. Our work demonstrates that smokers who switch exclusively to Vuse Vibe or Vuse Ciro ENDS products are exposed to significantly lower levels of HPHCs. The BoE decreases are very similar in magnitude to those observed with smoking abstinence. Switching to Vuse ENDS products provides nicotine for smokers without exposure to combustion-related toxicants. Similarly, the results reported here demonstrate that levels of select BoPH show rapid improvement following the switch to Vuse ENDS products, and those changes are concordant with those detected in smoking abstinence. While complete abstinence is the best option for reducing the harm from smoking, Vuse ENDS products may offer less harmful alternatives for smokers who are unwilling or unable to quit smoking, and may thus present less individual health risk to tobacco product consumers than cigarette smoking.

## Data Availability

The applicable data generated or analyzed during this study are included in this manuscript (and its Supplementary Materials). Additional dataset access for this study is administered through an internal Data Sharing Committee on reasonable request following completion of a data sharing request form and if applicable, a Data Access Agreement. Requests for data sharing in the first instance should be emailed to the corresponding author.

## Supplementary Materials

The following supporting information can be downloaded at: https://www.mdpi.com/article/10.3390/toxics11070564/s1, Inclusion/Exclusion Criteria for the clinical study population; Table S1: Characteristics of Vuse Vibe or Vuse Ciro Electronic Nicotine Delivery Systems (ENDS) products, Table S2: Demographics and baseline characteristics of study participants, Table S3: Harmful and potentially harmful constituents (HPHCs), Biomarkers of Exposure (BoE), and Bioanalytical Methods, Table S4: Consumption of e-liquid (grams) in Vuse Vibe and Vuse Ciro study groups, and Table S5: Summary of the most commonly reported adverse events and their relatedness to the study products. References [30,35,36,37,38,39] are cited in the main text.
