# Supplementary material for: Changes in Biomarkers of Exposure and Potential Harm in Smokers Switched to Vuse Vibe or Vuse Ciro Electronic Nicotine Delivery Systems
Source: Toxics. 2023 Jun 29;11(7):564. doi: 10.3390/toxics11070564 (PMC10384956; doi:10.3390/toxics11070564)
Supplement: Supplementary file 1 [file toxics-11-00564-s001.zip › toxics-2392936-supplementary.pdf]

Supplementary Section

Inclusion/Exclusion Criteria for the clinical study population

Inclusion Criteria

Subjects who met the following criteria were eligible for inclusion in the study:

1. Able to read, understand, and willing to sign an informed consent form (ICF) and complete questionnaires written in English;
2. Generally healthy males or females, 21 to 60 years of age (inclusive);
3. Screening expired-air carbon monoxide (ECO) level ≥ 15 parts per million (ppm):
4. Self-reports that cigarettes are the only tobacco or nicotine-containing product used within 30 days of the screening visit;
5. Self-reports, at the screening visit, smoking at least 10 cigarettes per day that are filtered, non-menthol, 83 mm to 100 mm length, and inhaling the smoke for at least 6 months prior to the screening visit;
6. Positive urine cotinine test at screening and enrollment;
7. Willing to switch from current cigarettes to either Vibe, Solo Gen 2, Ciro, or to abstain from smoking for 7 days during in-clinic confinement;
8. Females of childbearing potential must be willing to use a form of contraception acceptable to the Investigator from the time of signing the ICF until study discharge, or be surgically sterile for at least 90 days prior to the screening visit;
9. Able to safely perform the required study procedures, as determined by the Investigator.

Exclusion Criteria

The following excluded potential subjects from the study:

1. Clinically significant or unstable/uncontrolled acute or chronic medical conditions at screening, as determined by the Investigator, that would preclude a subject from participating safely in the study (e.g., hypertension, asthma or other lung disease, cardiac disease, neurological disease, or psychiatric disorders) based on screening assessments such as safety labs, medical history, and physical/oral examinations;
2. Self-reports or safety labs that indicate diabetes;
3. Self-reports of stomach ulcers;
4. At risk of heart disease, as determined by the Investigator;
5. Use of medicine for treatment of depression or asthma 30 days prior to screening;

6. Systolic blood pressure of ≥150 mmHg or a diastolic blood pressure of ≥95 mmHg, measured after being seated for 5 minutes;
7. Positive test for human immunodeficiency virus (HIV), hepatitis B surface antigen (HBsAg), or hepatitis C virus antibody (anti-HCV);
8. Hemoglobin level is < 12 g/dL at screening;
9. History or presence of hemophilia or any other bleeding disorders;
10. History or presence of clotting disorders with concomitant use of anticoagulants (e.g., clopidogrel [Plavix®], warfarin [Coumadin®, Jantoven®], aspirin [> 325 mg/day]) at least 30 days prior to screening; refer to Section 7.4: Concomitant Medications
11. Given a whole blood donation within 8 weeks (≤56 days) prior to enrollment;
12. Plasma donation within ≤ 7 days prior to enrollment;
13. Weight of ≤110 pounds;
14. Poor peripheral venous access;
15. Postponing a decision to quit smoking (defined as planning a quit attempt within 30 days of screening) or using tobacco-or nicotine-containing products to participate in this study;
16. Employed by a tobacco company, the clinical study site, or handles e-liquids or unprocessed tobacco as part of his/her job;
17. Use of any medication or supplement that aids in smoking cessation, including but not limited to any nicotine replacement therapy (NRT) (e.g., nicotine gum, lozenge, patch), varenicline (Chantix®), bupropion (Wellbutrin®, Zyban®), or lobelia extract within 30 days of the screening visit;
18. Use of injectable forms of medication(s), with the exception of injectable forms of birth control that are not required to be administered during the study period;
19. Self-reports drinking more than 14 servings of alcoholic beverages per week (1 serving = 12 oz of beer, 6 oz of wine, or 1.5 oz of liquor);
20. Females who have a positive pregnancy test, are pregnant, breastfeeding, or intend to become pregnant during the course of the study;
21. Females ≥35 years of age currently using systemic, estrogen-containing contraception, or hormone replacement therapy for menopause-related symptoms;
22. A positive urine drug screen without disclosure of the corresponding prescribed concomitant medication(s) at the screening visit or enrollment;
23. A positive alcohol breathalyzer test at screening or enrollment;
24. Regularly exposed to solvent fumes or gasoline (e.g., as a painter or auto mechanic);
25. Participation in another clinical study within 30 days prior to the time of consent. The 30-day window for each subject will be derived from the date of the last study event in the previous study to the time of consent in the current study;

26. Determined by the Investigator to be inappropriate for this study, including a subject who is unable to communicate or unwilling to cooperate with clinical staff;
27. Unable or unwilling to participate in the in-clinic confinement for the full study duration (a total of 10 days).

Supplementary Table S1: Characteristics of Vuse Vibe or Vuse Ciro Electronic Nicotine Delivery Systems (ENDS) products

ENDS Product Characteristics	Investigation Products	
	Vuse Vibe	Vuse Ciro
Type of system	Closed Tank	Cig-a-like
E-liquid capacity (mL)	1.9	0.9
Nicotine (% by weight)	3.0	1.5
Propylene glycol/glycerin ratio	20/80	29/71
Mean nicotine yield* (mg/puff)	0.09	0.05
Battery capacity (mAh)	≥ 550	≥ 260
Maximum puff duration (seconds)	6	10

Supplementary Table S1: Characteristics of Vuse Vibe or Vuse Ciro ENDS products: Key product characteristics of the Vuse ENDS products. *Machine smoking regime: 55 mL puff, 3 second duration, 30 second intervals.

Supplementary Table S2: Demographics and baseline characteristics of study participants

Parameter	Vuse Vibe (N = 37)	Vuse Ciro (N = 37)	Abstinence (N = 16)
Age (years): Mean (SD)	38.8 (11.1)	41.0 (11.0)	39.9 (8.0)
Weight (kg): Mean (SD)	85.0 (20.6)	82.5 (18.3)	83.2 (19.4)
Height (cm): Mean (SD)	172.7 (8.5)	173.2 (8.0)	172.2 (10.4)
BMI (kg/m2): Mean (SD)	28.3 (5.5)	27.6 (6.5)	28.0 (5.4)
Gender [n (%)]			
Male	25 (67.6%)	24 (64.9%)	9 (56.3%)
Female	12 (32.4%)	13 (35.1%)	7 (43.8%)
Smoking status			
Number of years smoked: Mean (SD)	23.3 (12.3)	25.8 (12.1)	24.4 (11.2)
Number of cigarettes smoked per day: Mean (SD)	17.3 (7.3)	15.9 (5.3)	18.3 (7.1)
Ethnicity [n (%)]			
Hispanic/Latino	1 (2.7%)	3 (8.1%)	0
Not Hispanic/Latino	35 (94.6%)	34 (91.9%)	16 (100.0%)
Not Reported	1 (2.7%)	0	0
White	29 (78.4%)	25 (67.6%)	11 (68.8%)
Black/African American	5 (13.5%)	9 (24.3%)	4 (25.0%)
Native Hawaiian/Other Pacific Islander	0	0	0
Asian	1 (2.7%)	0	0
American Indian/Alaska Native	0	2 (5.4%)	0
Multiple	2 (5.4%)	1 (2.7%)	1 (6.3%)

Supplementary Table S2: Demographic and baseline characteristics of study participants: Demographic and baseline smoking status of Vuse Vibe and Vuse Ciro study participants**.** Abbreviations: N = total number of participants randomized to a study group; SD = standard deviation; BMI = body mass index; n = number of participants available for a given observation.

Supplementary Table S3: Harmful and potentially harmful constituents (HPHCs), Biomarkers of Exposure (BoE), and Bioanalytical Methods

Tobacco constituent	Biomarker	Abbreviation	Matrix	Method	LOD	LLOQ
1-aminonaphthalene	1-aminonaphthalene	1-AN	Urine	GC-MS[37]	0.6 ng/L	1.8 ng/L
2-aminonaphthalene	2-aminonaphthalene	2-AN	Urine	GC-MS[37]	0.57 ng/L	1.7 ng/L
4-aminobiphenyl	4-aminobiphenyl	4-ABP	Urine	GC-MS[37]	0.51 ng/L	1.5 ng/L
o-Toluidine	*o*-toluidine	*o-Tol*	Urine	GC-MS[37]	0.83 ng/L	10 ng/L
Acrylonitrile	2-cyanoethyl mercapturic acid	CEMA	Urine	LC-MS/MS[36]	0.08 ng/mL	0.25 ng/mL
Crotonaldehyde	3-hydroxy-1-methylpropyl-mercapturic acid	HMPMA	Urine	LC-MS/MS[36]	0.49 ng/mL	5 ng/mL
Acrolein	3-hydroxypropyl mercapturic acid	HPMA	Urine	LC-MS/MS[36]	12.58 ng/mL	25 ng/mL
1,3-butadiene	Monohydroxybutyl mercapturic acid	MHBMA	Urine	LC-MS/MS[36]	0.029 ng/mL	0.129 ng/mL
Benzene	S-phenyl mercapturic acid	SPMA	Urine	LC-MS/MS[36]	5.0 pg/mL	0.02 ng/mL
Benzo[a]pyrene	3-hydroxy-benzo[a]pyrene	3-0H-B[*a*]P	Urine	LC-MS/MS[38]	33.3 fg/mL	100 fg/mL
4-(methylnitrosamino)-	4-(methylnitrosamino)-1-(3-pyridyl)-1-	NNAL	Urine	LC-MS/MS[335]	2 pg/mL	5 pg/mL

Tobacco constituent	Biomarker	Abbreviation	Matrix	Method	LOD	LLOQ
1-(3-pyridyl)-butanol)	butanol) + glucuronides					
N′-nitrosonornicotine	N′-nitrosonornicotine + glucuronides	NNN	Urine	LC-MS/MS[35]	0.8 pg/mL	2 pg/mL
Carbon monoxide	Carboxyhemoglobin	COHb	Whole Blood	HS-GC-MS[30]	0.40%	0.75%
Total nicotine equivalents						
Unconjugated Nicotine	Nicotine	Nic	Urine	LC-MS/MS[39]	0.5 ng/L	1.5 ng/L
Unconjugated Cotinine	Cotinine	Cot	Urine	LC-MS/MS[39]	0.17 ng/L	0.51 ng/L
Unconjugated trans-3′-hydroxycotinine	Nicotine	OH-Cot	Urine	LC-MS/MS[39]	0.21 ng/L	0.63 ng/L
Nicotine-*N*-glucuronide	Cotinine	Nic-Gluc	Urine	LC-MS/MS[39]	0.17 ng/L	0.51 ng/L
Cotinine-*N*-glucuronide	Nicotine	Cot-Gluc	Urine	LC-MS/MS[39]	0.33 ng/L	0.99 ng/L
Trans-3′-hydroxycotinine-O-glucuonide	Metabolite of trans-3'-hydroxycotinine	OH-Cot-Gluc	Urine	LC-MS/MS[39]	0.75 ng/L	2.25 ng/L

Supplementary Table S3: Harmful and potentially harmful constituents (HPHCs), Biomarkers of Exposure (BoE), and Bioanalytical Methods: A summary of HPHCs and the corresponding BoE quantified in the Vuse Vibe and Vuse Ciro study groups. Total nicotine equivalents are determined as a composite measure of nicotine and the five metabolites, as described in Materials and Methods. Abbreviations: GC = gas chromatography; MS = mass spectrometry; LC = liquid chromatography; HS = headspace; LOD = limit of detection; LLOQ = lower limit of quantification. The BoE and HPHCs were quantified by previously described bioanalytical methods: aromatic amines (1-AN, 2-AN, 4-ABP and *o-Tol*) [37]; mercapturic acids (CEMA, HMPMA, HPMA, MHBMA and SPMA) [36]; 3-0H-B[a]P [38]; TSNAs (NNAL and NNN) [35]; and nicotine exposure as determined by total nicotine equivalents [39]. CoHb was measured as described in Kanobe et al., 2022 [30].

Supplementary Table S4: Consumption of e-liquid (grams) in Vuse Vibe and Vuse Ciro study groups

Time Point	Vuse Vibe			Vuse Ciro		
	N	Mean±SD	Min, Max	N	Mean±SD	Min, Max
Day 1	36	0.82±0.52	0.08, 2.21	37	1.00±0.73	0.04, 2.57
Day 2	35	0.99±0.75	0.08, 3.23	37	1.16±0.84	0.14, 3.79
Day 3	35	1.04±0.69	0.11, 3.02	37	1.33±0.91	0.06, 3.69
Day 4	35	1.09±0.73	0.13, 3.31	37	1.46±1.02	0.06, 3.69
Day 5	35	1.07±0.72	0.07, 2.94	37	1.50±1.06	0.21, 4.28
Day 6	33	1.24±0.80	0.14, 3.44	36	1.53±1.01	0.18, 3.85
Day 7	33	1.29±0.90	0.15, 4.27	35	1.77±1.32	0.17, 6.20
Cartridges used daily (mean±SD)		0.72±0.36			1.76±1.01	

Supplementary Table S4: Consumption of e-liquid in Vuse Vibe and Vuse Ciro study groups: Daily usage of e-liquid (grams) in Vuse Vibe and Vuse Ciro study groups, and the mean daily usage of cartridges over 7 days in each product group. Abbreviations: N = total number of participants randomized to a study group; SD = standard deviation; Min = minimum; Max = maximum.

Supplementary Table S5: Summary of the most commonly reported adverse events and their relatedness to the study products

Adverse Event (AE)	Study Group				
	Vuse Vibe (N=37)		Vuse Ciro (N=37)		Abstinence (N=16)
	Total number of Reported AEs	Total Number of AEs Judged as 'Related' or 'Possibly-related'*	Total number of Reported AEs	Total Number of AEs Judged as 'Related' or 'Possibly-related'*	
Headache	4	3	3	1	1
Cough	4	4	2	3	1
Back pain	3	0	0	0	2
Oropharyngeal pain	3	2	3	3	0
Musculoskeletal pain	1	0	1	1	1
Nausea	3	2	0	0	0
Dry mouth	1	1	1	1	0
Dry throat	0	0	1	1	0

Upper respiratory tract infection	1	0	2	1	0

Supplementary Table S5: Summary of the Most Commonly Reported Adverse Events. *Adverse events that were judged by the Principal Investigator, as ‘related’ or ‘possibly related’ were those that followed a reasonable temporal sequence from use of the study products, and that could be excluded as being possibly caused by other factors. Abbreviation: N = total number of participants randomized to a study group
